# Phytochemical Analysis and Antidiarrheal Activity of Stem Bark Decoctions of *Pentadesma butyracea* Sabine (Clusiaceae)

**DOI:** 10.3390/molecules29235789

**Published:** 2024-12-07

**Authors:** Ericka Lorleil Mayindza Ekaghba, Manon Grenet, Pierrick Gandolfo, Corinne Loutelier-Bourhis, Isabelle Schmitz, Carlos Afonso, Patrice Lerouge, Line Edwige Mengome

**Affiliations:** 1 Institut de Pharmacopée et Médecines Traditionnelles (IPHAMETRA), Centre National de la Recherche Scientifique et Technique (CENAREST), Libreville BP 12 141, Gabon; ericka.mayindza-ekaghba@univ-rouen.fr; 2 Université de Rouen Normandie (UNIROUEN), Normandie Univeristy, GlycoMEV UR 4358, SFR Normandie Végétal FED 4277, Innovation Chimie Carnot, IRIB, GDR CNRS Chemobiologie, F-76000 Rouen, France; 3 Université de Rouen Normandie (UNIROUEN), Normandie Univeristy, Inserm U1245, F-76000 Rouen, France; manon.grenet@univ-rouen.fr (M.G.); pierrick.gandolfo@univ-rouen.fr (P.G.); 4 Université de Rouen Normandie, INSA Rouen, Normandie Univeristy, CNRS, UMR6014—COBRA, F-76000 Rouen, France; corinne.loutelier@univ-rouen.fr (C.L.-B.); isabelle.schmitz@cnrs.fr (I.S.); carlos.afonso@univ-rouen.fr (C.A.)

**Keywords:** *Pentadesma butyracea*, biflavonoids, toxicity, antioxidant, antidiarrheal agent

## Abstract

*Pentadesma butyracea* is a medicinal plant of which bark decoctions are used in traditional medicine for the treatment of diarrhea symptoms in Gabon. The aim of the present work was to perform phytochemical and pharmacological analyses of decoctions of *P. butyracea* bark. In a principal approach, spectrophotometric analyses were used to quantify phenolic compounds, followed by liquid chromatography coupled to mass spectrometry analysis that allowed the identification of flavanone–flavone dimers as the main metabolites. Pharmacological analyses showed the absence of toxicity, thus confirming the safety of use of this decoction in traditional medicine. The antioxidant activity of the bark decoctions was demonstrated to depend on their phenolic contents. The decoction of stem barks harvested during the rainy season also induced a dose-dependent relaxation of isolated ileum fragments from Wistar rats. In addition, the antidiarrheal activity of *P. butyracea* barks was investigated against castor oil-induced diarrhea. The oral administration of different concentrations of this decoction led to a decrease in wet stools, indicating an antidiarrheal effect at the doses that were used. These results encourage the deepening of bio-guided research on *P. butyracea* bark decoctions in order to propose standard traditional medical treatments.

## 1. Introduction

Ethnopharmacology has gained a considerable reputation, most notably in African and Asian countries. Ethnopharmacological research has become increasingly of interest for the development of bioactive phytochemicals as novel and effective preventive and therapeutic strategies for various diseases. However, to bridge the gap between local uses of medicinal plant extracts to a medication delivered as a pharmaceutical prescription, ethnopharmacology has to meet the standards of pharmacological research practises, which include pharmacological and clinical studies of traditional medicines and the identification and accurate quantification of metabolites relevant for a specific biological activity. Functional intestinal disorders involve chronic digestive symptoms indicating a dysfunction in the gastrointestinal tract without any evidence of an organic disease. The prevalence of functional intestinal disorders in Gabon was previously investigated in health care facilities [1]. This study revealed a frequency of 12.55%, mainly concerning women (63%) and an average age of 46 years. Abdominal pain and constipation were the most recorded clinical signs, and the most prevailing associated diseases were gastroesophageal reflux and hemorrhoids [1].

An ethnopharmacological and ethnobotanical study was carried out in Gabon by the Cultural and Technical Cooperation Agency (CTCA). These surveys were conducted in four major Gabonese towns (Libreville, Lambaréné, Franceville, and Oyem), and the medicinal plants used to treat various health symptoms are listed below. In this list, we have selected four plants used for the treatment of diarrhea symptoms, constipation, or abdominal pain because these pathologies appear in people suffering from irritable bowel syndrome (IBS) (Table 1). Among them, a stem bark decoction of *Pentadesma butyracea*, known for its antidiarrheal activities in traditional medicine in Gabon, was selected in the present study for phytochemical and pharmacological investigations.

*P. butyracea* is a large tree of dense forests belonging to the *Clusiaceae* family. It is present in forests from Sierra Leone to Gabon and Cameroon [2,3,4,5]. In these countries, a kind of butter is traditionally prepared from its seeds and, as a consequence, *P. butyracea* is known as the “tallow tree” or “butter tree”, “Krinda” in Côte d’Ivoire, “Abotoasebie” in Ghana, “Kpangnan” or “Sesseido” in Benin, and “Agnuhé” in Gabon [5]. This butter is used in traditional medicine as a massage oil for skin and hair care and in the manufacture of soap for its softening, lubricating, and healing qualities [4,6]. It has also been reported as able to delay skin ageing [7]. In Ghana, the root decoction is used to fight intestinal worms [8]. In Gabon, stem bark maceration is also used for treatment against skin parasites [9]. Extracts isolated from P. butyracea organs (stem barks, leaves, seeds, and roots) are also commonly used as traditional medical treatments of several diseases including breast pain and genitourinary system disorders [10,11]. With regard to previous phytochemical studies carried out on *P. butyracea*, various terpenes have been identified in essential oils [4,12], as well as xanthones and triterpenes in a methanol extract [13,14]. *P. butyracea* seeds also accumulate alkaloids, saponins, tannins, and phenolic metabolites [15].

The aims of the present work were to perform phytochemical and pharmacological analyses of *P. butyracea* stem bark decoctions, one of the plant extracts used in traditional medicine in Gabon for the treatment of diarrhea symptoms. Stem barks were harvested in the rainy and dry seasons from the same tree and from a young tree to investigate whether their extractable contents depend on seasons and age. Indeed, barks are indifferently collected all year round in Gabon for the preparation of decoctions from different trees. Phytochemical analysis of this extract was achieved by a spectrophotometric quantification of phenolic compounds and then by an identification of metabolites by ultra-high-performance liquid chromatography coupled to a quadrupole-Orbitrap mass spectrometer (UHPLC-ESI-MS/MS). Then, the in vitro and in vivo safety, antioxidant activity, and antidiarrheal potential of a *P. butyracea* stem bark decoction were investigated.

## 2. Results

### 2.1. Phytochemical Analyses of Decoctions of Pentadesma butyracea Stem Barks

#### 2.1.1. Total Phenolic Contents

The yields of *P. butyracea* decoctions obtained from stem barks collected during the dry season (DPBD), the rainy season (DPBR), and from a young tree during the dry season (DPBY) were, respectively, 6.24%, 8.43%, and 4.11% of the crude materials. The contents of phenolic compounds of these samples were estimated by spectrophotometry by measuring the reduction of the Folin–Ciocalteu reagent with gallic acid as standard. The total flavonoid contents of the decoctions of the *P. butyracea* stem barks were then investigated using the aluminium chloride method and quercetin as reference. The data on phenolic content were determined from a calibration curve (Y = 0.016X + 0.06309, R^2^ = 0.9916) of gallic acid expressed in gallic acid equivalents per milligram (GAE/mg) of dry extract (Table 2). The phenolic contents of the decoctions obtained from barks harvested during the dry season, i.e., DPBD and DPBY, were determined to be higher than those obtained from barks collected during the rainy season (DPBR) (129 ± 10.3 µg GAE/mg and 62.1 ± 1.4 µg GAE/mg, respectively, versus 44.1 ± 2.9 µg GAE/mg). The phenolic content of DPBD is three times higher than that of DPBR, although the harvesting was performed from the same tree. The results of flavonoid contents were obtained from the calibration curve (Y = 0.01458X − 0.04833, R^2^ = 0.9517) of quercetin expressed as quercetin equivalents per milligram (QE/mg) of dry extract (Table 2). The decoctions prepared from the same tree in both seasons (DPBD and DPBR) are the highest (150.3 ± 30.3 µg QE/mg and 83.0 ± 9.3 µg QE/mg, respectively). These results suggest that the accumulation of phenolic contents in *P. butyracea* stem barks depends on the harvesting season and on the age of the tree.

#### 2.1.2. Mass Spectrometry Identification of Biflavonoids in the Decoction of *Pentadesma butyracea* Stem Barks

Phytochemical analysis of the *P. butyracea* stem bark decoctions was performed by mass spectrometry on the DPBR sample. The metabolites were analyzed by ultra-high-performance liquid chromatography coupled with a quadrupole-Orbitrap mass spectrometer (UHPLC-ESI-MS/MS) in negative and positive ionic modes. In addition, the DPBR sample was submitted to methanolysis and trimethylsilylation to convert free or *O*-linked monosaccharides, acids, and phenolic compounds into their methylester or methylglycoside trimethylsilyl derivatives, which were then identified by gas chromatography coupled to electron ionization mass spectrometry (GC-EI-MS) (Table S1).

**Figure 1:**
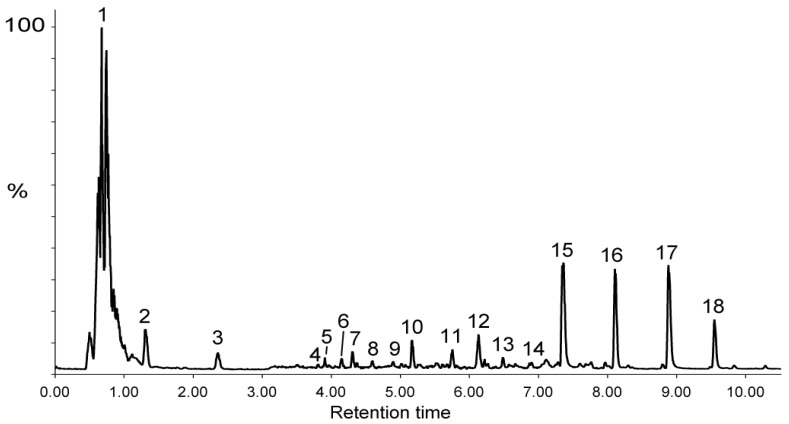
Total ion current chromatogram for the LC-ESI (−)-MS of the DPBR sample. Peak numbering refers to the main metabolites identified by analysis of their MS/MS fragmentation patterns and reported in Table 3.

Figure 1 shows the total ion current chromatogram for UHPLC-ESI-MS in the negative mode and the peak numbering assigned to the main metabolites annotated by LC-ESI-MS/MS analysis and reported in Table 3. The metabolite annotation was based on the accurate mass measurements in both the negative and positive ionization modes and their MS/MS fragmentation patterns in negative mode by comparison with the literature and databases (Massbank, PubChem, HMDB). In the negative ion mode, metabolites were detected either as a deprotonated molecule [M − H]^−^ or as a formate adduct ([M + HCOO]^−^). The elution peak between 0.5 and 1 min mainly contains small metabolites and sugars (compounds **1a** to **1n** in Table 3). For instance, [M − H]^−^ is assigned to 4-(4-deoxy-β-D-gluc-4-enuronosyl)-galacturonate, resulting from the degradation of cell wall pectins by pectate lyase. In addition, LC peaks 2 and 3 are assigned to citrate and methyl citrate, respectively.

**Figure 2:**
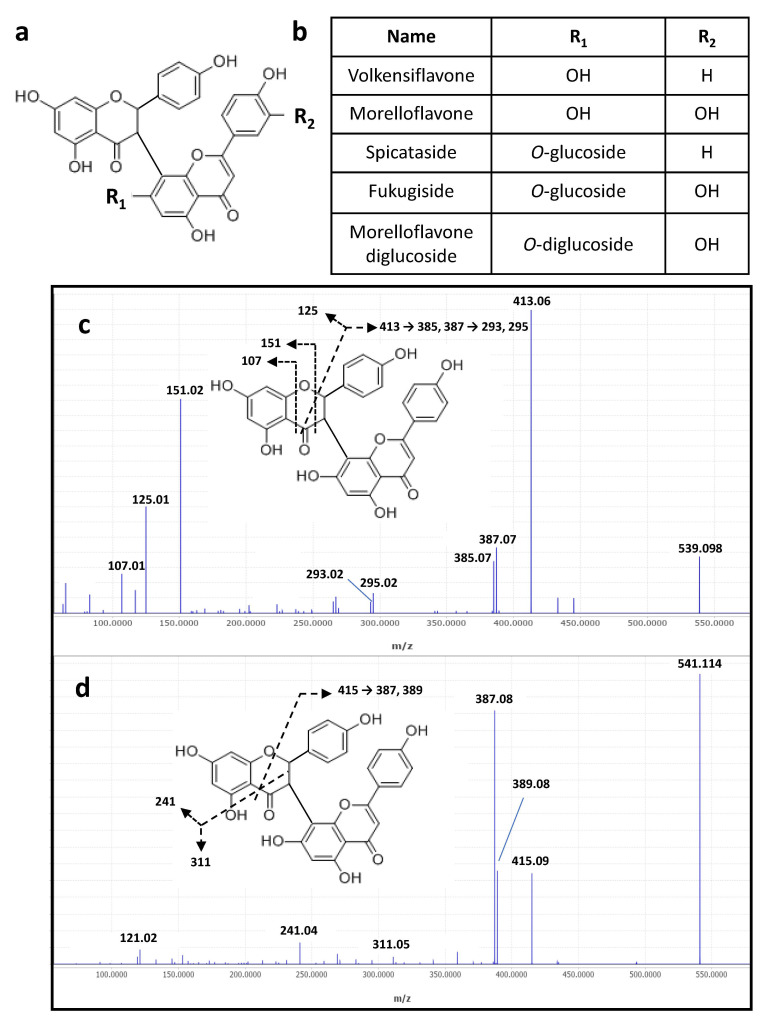
Proposed structures (**a**) and names (**b**) of biflavonoids annotated in the DPBR sample. ESI-MS/MS spectra of [M − H]^−^ (**c**) and [M + H]^+^ (**d**) of the flavanone–flavone dimer volkensiflavone at *m*/*z* 539.098 and 541.114, respectively.

The main metabolites (n° 15–18, Figure 1 and Table 3) of the DPBR sample were determined as being flavanone–flavone dimers, namely volkensiflavone and morelloflavone, and their respective mono- and diglucosides (Figure 2a,b). All together, these biflavonoids represented about 75% of the metabolites detected in the MS negative mode (Figure 2). Their negative MS/MS fragmentation patterns were consistent with published data on flavanone–flavone dimers [16] and on flavonoids [17]. For instance, MS/MS fragmentation of the [M − H]^−^ of volkensiflavone at *m*/*z* 539.098 (C_30_H_21_O_10_) resulted in the loss of 126 Da, yielding an intense fragment ion at *m*/*z* 413.06 (Figure 2c). This fragment arose from the cleavage of the pyranose ring of the flavanone moiety of the biflavonoid. Other diagnostic ions mainly resulted from the secondary fragmentation of this major ion. For morelloflavone ([M − H]^−^at *m*/*z* 555.094) and morelloflavone mono- and diglucoside ([M − H]^−^ at *m*/*z* 717.146 and *m*/*z* 879.199), a shift of 16 Da was observed for the main fragment ions. This is due to the presence of a luteolin instead of an apigenin flavone motif in these flavanone–flavone dimers. With regard to the mono- and diglycoside derivatives, the sugar composition determined by GC-EI-MS analysis indicated that glucose is the main hexose identified in the DPBR sample (Table S1). We thus postulated that the main glycoside derivatives were mono- and diglucosides, namely spicataside and fukugiside, respectively (Table 3 and Figure 2b). The same conclusions were drawn on the basis of the investigation of the MS/MS fragmentation patterns of [M + H]^+^ ions of biflavonoids in the MS positive mode (Table 3 and Figure 2d).

In addition to biflavonoids, less abundant metabolites were eluted between 3.79 and 6.13 min (Figure 1). Among them, the presence of vitexine-2-*O*-rhamnoside was deduced from the MS/MS spectrum of the [M − H]^−^ at *m*/*z* 577.156 (C_27_H_30_O_14_) in accordance with the literature data [18] and the identification of rhamnose as the main deoxyhexose in the decoction (Table 3). Other metabolites were identified as deoxyhexosyl hexose disaccharide linked to phenolic or benzoic derivatives (metabolites 5, 10, 11 and 14). These benzoic acids were also detected in the GC-EI-MS analysis (Table S1). The deoxyhexosyl hexose disaccharide motif was proposed as being a rhamnosylglucoside sequence, also called rutinoside, on the basis of the sugar composition of the DPBR sample (Table S1) and in agreement with the fragmentation patterns of homologous metabolites reported in the literature [19]. Among these rhamnosylglucoside-containing metabolites, we identified antiarol rutinoside, which was previously reported in plant extracts [20].

It is also worth noting that [M−H]− ions at m/z 371.098, 387.093 and 417.103 detected by UHPLC-ESI-MS/MS were assigned to benzoyl derivatives of polyhydroxy nonanedioic acid (Table 3). This diacid, also detected in the LC elution peak as a free diacid, could correspond to pentahydroxy 2, 3, 4, 6, 7 nonanedioic acid on the basis of its ESI-MS/MS fragmentation pattern (Figure S1b) and the EI-MS of its trimethylsilyl dimethylester derivative (Figure S1a). Its structure was also confirmed from the ESI-MS/MS fragmentation pattern of its 2-benzoyl derivative (metabolite 8, Table 3), as depicted in Figure S1c.

#### 2.1.3. Evaluation of Biflavonoid Contents in the DPB Samples

**Figure 3:**
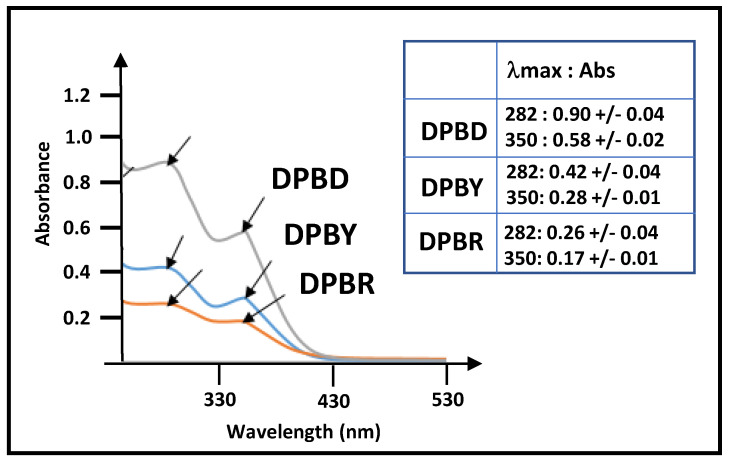
UV–visible spectra recorded between λ = 230 nm and 550 nm of 0.1 mg/mL solutions of decoctions of *P. butyracea* stem barks (*n* = 3). Absorbances of the different decoctions at λ_max_ = 282 and 350 nm are reported in the inserted table.

Flavanone–flavone dimers, such as volkensiflavone and morelloflavone, exhibit specific UV absorbances at λ_max_ = 282 and 350 nm due to flavone and flavanone motifs [16]. Similar UV profiles and λ_max_ were observed in the UV spectra of decoctions of *P. butyracea* stem barks, indicating that mainly bioflavonoids (Figure 3), which represent about 75% of phenolic metabolites of the extract (Table 3), contribute to UV absorbances at these wavelengths, although we cannot rule out the contribution of minor phenolic compounds to these UV profiles. As a consequence, a quantification of biflavonoids at λ_max_ = 282 and 350 nm was performed to investigate the biflavonoid contents in the decoctions of *P. butyracea* stem barks from young or adult *P. butyracea* trees or between stem barks collected during either the dry or rainy season (Figure 3). In accordance with data on phenolic contents determined by quantification through a spectrophotometric assay (Table 2), UV profiles confirmed that the decoction of *P. butyracea* stem barks collected during the dry season (DPBD) contains higher amounts of biflavonoids than samples collected during the rainy season (DPBR) or from young trees (DPBY).

### 2.2. Pharmacological Analyses of Decoction of P. butyracea Stem Barks

#### 2.2.1. Antioxidant Activity Assay

The DPPH radical scavenging activities of decoctions of stem barks from *P. butyracea* are presented in Table 4. Extracts from decoctions prepared from barks harvested during the dry season (DPBD and DPBY) presented a higher antioxidant activity than the decoction obtained from barks collected during the rainy season (DPBR), with IC_50_ values of 8.1 ± 0.6, 11.0 ± 2.0, and 23.5 ± 2.1 µg/mL, respectively. All data were compared with the IC_50_ value of standard ascorbic acid (6.2 ± 1.2 µg/mL).

#### 2.2.2. Toxicity Assays

To examine the putative cytotoxic activity of DPBR, we first used two human cell lines, HEK-293 and hCMEC/D3, obtained from an embryonic kidney and adult cerebral vessels, respectively. We found that the incubation of both cell lines with graded concentrations of DPBR had no impact on cell viability, even at a high concentration (100 µg/L) and during a 48 h incubation period (Figure S2).

The DPBR sample was also evaluated for acute oral toxicity in Wistar rats. Over a 14-day period, the DPBR sample did not exhibit any toxicity or behavioural changes in animals that received at a single dose of 2000 mg/kg of body weight. Moreover, we did not observe any weight loss over two weeks of observation (Figure S3). We thus concluded that DBPR does not induce any oral acute toxicity, even at a high dose.

#### 2.2.3. Effect of DPBR Sample on Smooth Muscle and Antispasmodic Activity 

The effect of different doses of DPBR on the contractile activity of rat ileal smooth muscle was assessed in vitro to study their impact on spontaneous ileal contractions. Loperamide was used as a positive control. As reported in Table 5, DPBR was found to induce a dose-dependent decrease in the contractile activity. The DPBR decoction tested at 4 mg/mL totally relaxed the smooth muscle (100% relaxation). This showed that DPBR exhibits spasmolytic activity resulting from the muscle-relaxing properties of metabolites in this plant extract.

To check the effect of DPBR on the cholinergic system, the same concentrations of the extract were applied after the administration of acetylcholine, a chemical neurotransmitter. After pre-contraction of the smooth muscle with acetylcholine, DPBR was added at doses ranging from 1 to 4 mg/mL. The percentage of relaxation on the smooth muscle increased from 0 to 42.9% (Table 5). DPBR is thus able to antagonize the smooth muscle-stimulating action of acetylcholine. This shows that the DPBR extract has an antispasmodic effect.

#### 2.2.4. In Vivo Antidiarrheal Activity of DPBR

We then investigated the in vivo antidiarrheal activity of DPBR on castor oil-induced diarrhea and enteropooling in Wistar rats to corroborate the ethnobotanical information reported on stem barks of *P. butyracea* in Gabon. The antidiarrheal activity of DPBR at doses ranging from 100 to 1000 mg/kg of body weight was then evaluated in vivo in rats in the castor oil-induced diarrhea model and compared to loperamide at 5 mg/kg, which was used as a positive control. The DPBR sample showed 100% protection against diarrhea at a concentration of 500 mg/kg of body weight (Table 6). In addition, the moisture content dropped down from 75 to 39% at the same dose and the inhibition of diarrhea was 100%. These data suggest that this decoction induces the antisecretory mechanisms of water and electrolytes, and that the dose of 500 mg/kg can be taken as the reference dose for antidiarrheal treatment with this decoction. At the higher dose of 1000 mg/kg of the DPBR sample, the percentage of inhibition of defecation largely decreased, which is likely due to an inhibitory response from the decoction. The dose of the decoction that induces 50% of humidity (EC_50_) was calculated as being 297 ± 33 mg/kg.

The effects of DPBR on castor oil-induced enteropooling are presented in Table 7. DPBR induced a major decrease in the intestinal fluid volume in a dose-dependent manner compared to the negative control (distilled water). At doses of DPBR ranging from 100 to 500 mg/kg, the mean weight of intestinal content decreased from 2.7 g to 1.1 g, against 3.2 g for the control rat group. These results show that DPBR exhibits a relevant effect on the accumulation of intestinal fluid volume induced by castor oil administration.

## 3. Discussion

Plant compounds are widely used in traditional medicine for their healing power [21]. With regard to *P. butyracea*, previously reported phytochemical studies allowed the identification of terpenes in essential oils of seeds and leaves [4,12] and xanthones and triterpenes in a methanol extract [13,14]. *P. butyracea* seeds also accumulate alkaloids and phenolics [15]. The presence of coumarins, tannins, flavones, sterols, and saponins was also reported in *P. butyracea* leaf decoctions [4]. The present study focuses on the phytochemical content, the safety, and the antioxidant, smooth muscle relaxation, and antidiarrheic activities of stem bark decoctions of *P. butyracea,* which are used in traditional medicine as an antidiarrheal agent.

Various analytical techniques are usually performed for the identification of phytochemicals [22]. In our study, the spectrophotometric analysis of the bark decoctions of *P. butyracea* first showed that polyphenols are major metabolites. The analyses of DPBR by GC-EI-MS allowed the identification of its constitutive monomers. Then, UHPLC-ESI-MS/MS analysis revealed that the flavanone–flavone biflavonoids represent about 75% of the metabolites present in this decoction. It is worth noting that biflavonoids have been previously reported in *Pentadesma grandifolia* [14]. Rhamnosylglucoside-containing metabolites were also identified by ESI-MS/MS, as well as benzoyl derivatives of polyhydroxy nonanedioic acid that have never been reported in the literature to date.

Although phenolic compounds are widely used in human health, it remains crucial to evaluate their toxicological risk. The putative toxicity of the DPBR sample was first investigated in vitro on cell cultures. Our results showed that DPBR did not affect the cell survival of two different cell lines (Figure S2). Moreover, the feeding of Wistar rats with up to 2000 mg/kg of body weight did not reveal any acute toxicity from DPBR (Figure S3). The oral administration of the decoction of *P. butyracea* was thus considered as safe. As a consequence, the absence of toxicity of *P. butyracea* bark decoctions could justify their use in traditional medicine. This result is consistent with previous studies reporting the absence of acute toxicity from hydroalcoholic extracts of *P. butyracea* seeds and leaves [11,23].

Antioxidant metabolites are important for human health for their ability to neutralize free radicals. Studies on various *P. butyracea* extracts have reported that antioxidant activities are correlated with their high phenolic contents [11,15,24]. This is corroborated by our results, which show that the extract richest in polyphenols, DPBD, has the highest antioxidant activity. This is also in accordance with its content of biflavonoids, estimated by UV spectrophotometry, in comparison to DPBY and DPBR (Figure 3). The antioxidant activity of biflavonoids has previously been reported [16,25,26] and arises from the radical scavenging activity of their phenolic motifs [27,28]. It is worth noting that oxidative stress could have direct or indirect effects on gastrointestinal tract responses [29,30].

In vitro evaluation of contractile activity showed that DPBR exhibits relaxing effects on the smooth muscle of rats in a dose-dependent manner [31] and is able to antagonize the smooth muscle-stimulating action of acetylcholine. The alteration of smooth muscle contractility is among the key mechanisms involved in the pathophysiology of gastrointestinal disorders. The myorelaxant and antispasmodic activities of *P. butyracea* bark decoctions on smooth muscle have not yet been studied. Several studies have previously reported on the effects of plant extracts on smooth muscle activity [32,33,34,35,36,37]. It should be noted that biflavonoids isolated from *Allanblackia floribunda* have also been shown to exhibit vasorelaxing activities [24].

Castor oil is able to produce diarrhea symptoms by releasing ricinoleic acid, which causes local irritation and inhibition of the intestinal mucosa, resulting in the release of prostaglandins that induce gastrointestinal motility and the secretion of water and electrolytes [38,39]. Diarrhea occurs when there is a disturbance in the motility of the smooth intestinal muscles that leads to a water imbalance in the gastrointestinal tract [40]. In our study, the antidiarrheal effect of DPBR at doses ranging from 100 to 500 mg/kg of body weight significantly delayed the diarrheal onset and decreased the frequency of defecation and weight of feces in a dose-dependent manner. Protection reached 100% at a dose of 500 mg/kg of body weight with ED_50_ = 297 ± 33 mg/kg. At doses ranging from 100 to 500 mg/kg, DPBR also caused a major decrease in the accumulation of the intestinal fluid volume after castor oil administration.

Loperamide was used as a positive control that antagonizes the action of castor oil due to its anti-motility and anti-secretion properties [41]. Given the mechanisms of action of castor oil, several mechanisms may be involved in the observed antidiarrheal activity. DPBR could inhibit cyclooxygenase, which reduces prostaglandin production or the activation of Na^+^/K^+^-ATP-ase channels, which inhibits diarrhea by increasing normal fluid absorption; DPBR may also interfere with the action of adenylate. These results show that the DPBR extract exerts antidiarrheic activity by inducing antisecretory mechanisms that increase normal fluid absorption. In the castor oil-induced diarrhea model, the agents that inhibit diarrhea, using the mentioned mechanisms above, are considered to have antidiarrheal activity [37,42].

In our in vivo antidiarrheal assay, the percentage of inhibition of defecation and moisture largely decreased at a high concentration of the DPBR sample (1000 mg/mL) compared to lower doses. This is likely due to a phenomenon of hormesis, which is characterized by an inverted U-shaped dose–response relationship with a stimulating response to small doses but an inhibitory response at high doses [43]. This phenomenon of hormesis was reported in previous studies on the antidiarrheal activity of plant extracts [44,45].

## 4. Materials and Methods

### 4.1. Equipment

An OHAUS Adventurer balance was used for weighing, and the grinding of barks was performed using a Reisch:AEG typ: AM 80 NX2 industrial grinder. A CHRIST Alpha 1-2 LDplus freeze dryer was used to dry the extracts. Spectrophotometric analysis was conducted using a UV-VIS spectrophotometer (Drawell, Chongqing, China). Ultra-high-performance liquid chromatography coupled to mass spectrometry (UHPLC-ESI-MS/MS) analyses were performed using a UHPLC system (Vanquish, Thermo Scientific, San Jose, CA, USA) coupled to a quadrupole-Orbitrap mass spectrometer (Exploris 120, Thermo scientific) equipped with an electrospray ionization source. Gas chromatography coupled to an electron impact mass spectrometer (GC-EI-MS) was performed on an Agilent 8860 GC instrument coupled to a 5977-mass selective detector (MSD) quadrupole MS instrument (Agilent Technologies, Palo Alto, CA, USA). The RIKADENKY organ isolation device was used to assess the effect of plant extracts on smooth muscle; a dissection kit was used to isolate the organs.

### 4.2. Plant Material

*Pentadesma* butyracea Sabine stem barks were collected in 2020 in Libreville (Gabon). They were authenticated by Raoul Niangadouma and Nick Jordan Koumba at the National Herbarium of Gabon (NHG), where a sample is conserved as a reference. In the laboratory, these materials were kept in a glass bell. Two harvests were made from the same tree, one in the rainy season (R), the other in the dry season (D), and another from a young tree in the dry season (Y).

### 4.3. Chemicals

2,2-di(4-tert-octylphenyl)-1-picrylhydrazyl free radical (DPPH), Folin–Ciocalteu reagent, disodium hydrogen phosphate (Na_2_HPO_4_), monobasic potassium phosphate (KH_2_PO_4_), dimethyl sulfoxide (DMSO), quercetin, gallic acid, and ascorbic acid were purchased from Sigma Chemical Co. (St. Louis, MO, USA). Organic solvents, acids, and other chemicals such as ethanol, methanol, hydrochloric acid, aluminium chloride, sodium carbonate, sodium chloride, and potassium chloride were purchased from Merck (Darmstadt, Germany). All reagents and chemicals were of analytical grade and the organic solvents were of HPLC grade. All substances were stored in glass containers at room temperature.

### 4.4. Animal and Cell Line Model

Adult Wistar rats were from the animal house of the Institute of Pharmacopoeia and Traditional Medicine (IPHAMETRA), Libreville, Gabon. These animals were fed with industrial pellets containing 29% protein and had access to drinking water. All tests were carried out according to protocols already approved by the Department of Pharmacology and Toxicology of IPHAMETRA (agreement N° 001; MESRSTT/IPHAMETRA) and met international standards for animal studies [46]. Human cells were embryonic kidney HEK-293 cells (ATCC^®^, CRL-1573™, Manassas, VA, USA) and the human cerebral endothelial cell line (hCMEC/D3; kindly provided by Dr. Pierre-Olivier Couraud, Institute COCHIN, Paris, France).

### 4.5. Preparation of P. butyracea Stem Bark Decoctions

The stem barks were dried at the Department of Traditional Medicine of IPHAMETRA, Libreville, Gabon, for two weeks and then reduced to a fine powder using a grinder. Five hundred grams of the ground material was placed in a volume of 2 L of distilled water, brought to the boil at 100 °C, and stirred for 1 h. The aqueous solute was filtered, frozen, freeze-dried, and named according to the collection period and age of the tree, as follows: DPBR (rainy season), DPBD (dry season), and DPBY (young tree). The extract yields were calculated using the ratio of the mass of the decoction extract to the ground material. For bioassays, decoctions were then solubilized in 1% DMSO in water.

### 4.6. Determination of Total Phenolic Contents

The total phenolic contents (TPCs) of the decoctions of *P. butyracea* stem barks were determined by measuring the reduction of the Folin–Ciocalteu reagent into a blue solution by complexation of phenolic compounds of the samples [47]. Briefly, 1.5 mL of a Folin–Ciocalteu solution (10% in distilled water) was mixed with 500 µL of bark decoctions (1 mg/mL in distilled water) and then allowed to stand for 10 min. Afterward, 2 mL of 7.5% (*w*/*v*) sodium carbonate solution was added to each tube and kept at 37 °C for 1 h in the dark. Then, the absorbance of the respective solutions was determined at λ = 760 nm on a UV-VIS spectrophotometer, with the reaction mixture (water + 10% Folin solution + 7.5% sodium carbonate) as a blank. Concentrations of gallic acid from 10 to 60 µg/mL were used to draw a standard calibration plot. The TPCs of the decoctions were estimated as micrograms of equivalent gallic acid (GAE) per milligram of extract (µg gallic acid/mg). The following formula was applied to calculate the total concentration of phenolic content: TPC = P × V/m, where P is the gallic acid concentration in mg/mL, V is the volume (mL) of the sample used in the extraction, and m is the weight of the pure dried sample used (mg). All tests were carried out in triplicate.

### 4.7. Determination of Total Flavonoid Contents

The total flavonoid contents (TFCs) of the decoctions of *P. butyracea* stem barks were determined using the aluminium chloride (AlCl_3_) method [48]. Briefly, 1 mL of decoctions at 1 mg/mL in distilled water or standard quercetin solution (1 mL, 10 to 40 µg/mL) was added to test tubes containing 500 µL of 2% AlCl_3_ in methanol. The solutions were mixed properly and the tubes were kept at room temperature for 1 h. The appearance of a yellow colour indicated the presence of flavonoids. The absorbance was measured at λ = 430 nm against the reaction mixture (methanol + 2% AlCl_3_) as a blank. The TFCs were estimated as micrograms of equivalent of quercetin (QE) per milligram of extract (µg quercetin/mg) using the equation below to estimate the total flavonoid content: TFC = F × V/m, where F represents the quercetin concentration (µg/mL), V is the volume (mL) of sample used in the extraction, and m represents the weight of the pure dried sample used (mg). All tests were carried out in triplicate.

### 4.8. Gas Chromatography Coupled to an Electron Impact Mass Spectrometer (GC-EI-MS)

For an analysis of metabolites of the decoctions of *P. butyracea* stem barks by gas chromatography coupled to electron ionization mass spectrometry (GC-EI-MS), 1 mg of the sample was first submitted to methanolysis by heating the sample in 1 M HCl in methanol at 80 °C overnight to convert monosaccharides and phenolic compounds into their *O*-methyl glycosides/esters. After the evaporation of the methanol-HCl solution, the samples were then trimethylsilylated by heating for 20 min at 110 °C in hexamethyldisilazane/trimethylchlorosilane/pyridine (3:1:9). After the evaporation of the reagent, the samples were dissolved in cyclohexane before being analyzed by GC-EI-MS, which was performed on an Agilent 8860 GC instrument coupled to a 5977-mass selective detector (MSD) quadrupole MS instrument (Agilent Technologies, Palo Alto, CA, USA). Separations were carried out on a CP-Sil 5CB capillary column (Agilent Technologies) with a film thickness of 250 μm. The carrier gas was 99.9% helium at a flow rate of 1.3 mL·min^−1^. The injector and ion source temperatures were set to 280 and 230 °C, respectively. The samples were injected in 1:15 split mode. The temperature of the GC oven was first maintained at 40 °C for 3 min and then increased up to 160 °C at a rate of 15 °C·min^−1^, then up to 280 °C at a rate of 1.5 °C.min^−1^. For electron impact mass spectrometry (EI-MS), the ionization energy was 70 eV. Acquisitions were performed in full scan mode over a 50–550 mass range with a solvent delay time of 3 min. GC-EI-MS analyses were carried out in triplicate.

### 4.9. Ultra-High-Performance Liquid Chromatography Coupled to a Quadrupole-Orbitrap Mass Spectrometer (UHPLC-ESI-MS/MS)

A total of 5 mg of each sample was dissolved in 1 mL of HPLC-grade water and then filtrated through 0.5 mL centrifugal filters Ultracel 10 kDa (Amicon, Sigma Chemical Co., St. Louis, MO, USA) to remove high-molecular-weight compounds and impurities. The UHPLC-ESI-MS/MS analyses were performed using a UHPLC system coupled to a quadrupole-Orbitrap mass spectrometer equipped with an electrospray ionization source. The chromatographic separations were performed using a C18 silica-based column (Acquity UPLC HSS T3, 1.8 µm, 1.0 mm × 100 mm, Waters Corporation, Milford, MA, USA) with a prefilter of 0.2 µm, and kept at 50 °C during the analysis. The solvents (water and acetonitrile) were LC–MS grade (Fisher Chemical Optima, Illkirch, France). The formic acid was from LiChropur (Merck). An autosampler kept the samples at 6 °C. The injection volume was 3 µL. The solvents used for gradient separation were 0.1% (*v*/*v*) formic acid in water as mobile phase A and 0.1% (*v*/*v*) formic acid in acetonitrile as mobile phase B. The flow rate was 0.4 mL/min. The elution gradient was first 1% B for 1 min, then increased linearly to 100% B over 20 min, and then maintained at 100% B for 8 min. Samples were analyzed in both negative and positive modes. The ESI source parameters were as follows: spray voltages of 3500 V and 3000 V for positive and negative modes, respectively; sheath gas—35 (arbitrary unit), auxiliary gas—10 (arbitrary unit); sweep gas—2 (arbitrary unit); ion transfer tube—320 °C; and a vaporizer temperature of 275 °C. Data-dependent acquisitions were carried out in both positive and negative modes. The MS1 resolution was set to 60,000 with a standard AGC target, maximum injection time set to auto, microscan set to 1, RF lens set to 70%, and scan range set from *m*/*z* 80 to 1200. The EASY-IC internal standard was used. For MS/MS, the resolution was set to 15,000 with a maximum injection time of 50 ms. The isolation window was 2 *m*/*z*, dynamic exclusion was set to 4 s, mass tolerance was ±2 ppm, and the precursor intensity threshold was set to 5.10^5^ in the positive mode and 1.10^5^ in the negative mode. The HCD collision energies were 15%, 40%, and 60% in both positive and negative ion modes. Data processing was carried out using MZmine 2 (version 2.53). Annotation was performed based on accurate mass measurements and MS/MS spectra according to the literature data.

### 4.10. UV-VIS Spectroscopy

Solutions of 1 mg/mL in water of DPB decoctions were centrifuged at 3000 rpm for 10 min and filtrated through a Whatman n° 1 filter paper (Maidstone, UK). The samples were then diluted to 1:10 and scanned at wavelengths ranging from λ = 230 to 500 nm using a UV-VIS spectrophotometer (Drawell, Chongqing, China). Spectra were recorded in triplicate.

### 4.11. Antioxidant Assay

The free radical scavenging activity of the decoctions of *P. butyracea* stem barks was determined by the 2,2-di(4-tert-octylphenyl)-1-picrylhydrazyl (DPPH) method with some modifications [49]. Briefly, 1 mL of 2.5 mM DPPH in methanol was combined with 1 mL of decoctions at concentrations ranging from 50 to 1000 µg/mL. The mixture was shaken and then incubated for 1 h in the dark at room temperature. The absorbance was then measured at λ = 517 nm. Ascorbic acid was used as reference and the percentages of DPPH radical scavenging activity were calculated using the following formula: % inhibition DPPH = [Ac − As)/Ac] × 100, where Ac represents the absorbance of the blank containing methanol and DPPH (*v*:*v*) and As represents the absorbance of the samples containing DPPH and extracts or reference. The IC_50_ was calculated by plotting the percentage of radical scavenging activity against different concentrations of the sample using nonlinear regression with Graph Pad Prism version 8.4.3.686. All assays were carried out in triplicate.

### 4.12. In Vitro Cytotoxicity Assay

Human embryonic kidney HEK-293 cells (ATCC^®^, CRL-1573™) were cultivated in Dulbecco’s modified Eagle’s medium (DMEM, Gibco, Waltham, Massachusetts, USA) completed with fetal bovine serum (FBS 10%, Eurobio-Scientific, Les Ulis, France), antibiotic–antimycotic solution (penicillin/streptomycin/fungizone, 1%, Sigma Chemical Co., St. Louis, MO, USA), and sodium pyruvate (1%, Gibco). The human cerebral endothelial cells (hCMEC/D3) were cultivated in flasks previously coated with collagen I (50 µg/mL in sterile phosphate-buffered saline (PBS), 1 h, 37 °C) in EndoGroTM MV medium (Merk Millipore, containing 5% FBS, 5% glutamine, 0.2% EndoGRO-LS nutrient supplement, 0.1% epidermal growth factor, 0.1% hydrocortisone, 0.1% heparin sulphate, and 0.1% ascorbic acid). Both HEK-293 and hCMEC/D3 cell lines were maintained at 37 °C and 5% CO_2_ in a humidified atmosphere.

For the cell survival assay, HEK-293 cells (20,000 cells/well) and hCMEC/D3 (10,000 cells/well) were placed in a white flat-bottom 96-well plate (Corning, Boulogne-Billancourt, France) previously coated with poly-D-lysine (Corning) (30 µM in H_2_O, 1 h, 37 °C) or collagen I (50 µg/mL in PBS, 1 h, 37 °C), respectively. After 24 h, cells were rinsed with Dulbecco’s PBS (dPBS, Sigma Chemical Co., St. Louis, MO, USA) and incubated for 6, 24, or 48 h in an FBS-free medium in the absence or presence of graded concentrations of DPBR (1, 10, or 100 µg/mL). After incubation, the Cell Titer-Glo^®^ luminescent Cell Viability Assay (Promega, Madison, WI, USA) was used to quantify cell viability following the manufacturer’s protocol. The luminescence was measured using the InfinitePro200 plate reader (TECAN, Neuville-sur-Oise, France). Cytotoxicity assays were carried out in triplicate.

### 4.13. Acute Toxicity Assay

Wistar rats were subjected to an evaluation of acute toxicity induced by the DPBR sample according to established OECD 423 guidelines [50] and the *Guide for the Care and Use of Laboratory Animals* [46] with some modifications. A prior approval agreement (N° 001 MERSTT/IPHAMETRA) was obtained from the Ethics Committee on the Use of Animals of the Institute of Pharmacopoeia and Traditional Medicine (IPHAMETRA). The animals were divided into two groups of six (three males and three females) and fasted for 24 h before the experiment. The tested Wistar rats received the DPBR sample orally at a single dose of 2000 mg/kg. Control animals received only distilled water, and all were kept in the same environmental conditions. The animals were strictly observed for physiological symptoms such as weight loss, diarrhea, tremor, lethargy, and paralysis periodically for the first 4 hours during the 72 h period, after which point they were checked per day for 14 days for any lethality.

### 4.14. In Vitro Evaluation of the Contractile Activity and Antispasmodic Effect on Excised Ileum Fragments

The evaluation of the contractile and antispasmodic activities of DPBR was performed according to the literature data with some modifications [33,51]. Pieces of ileum were taken from the Wistar rats and preserved during the tests in Mac Ewen’s physiological solution. Fragments measuring 0.5 to 0.9 cm were fixed in a tank, called a survival tank, in an aerated thermostatic bath at 37 °C. The basic activity (ileum contractions) of the organ or stimulation of the organ with acetylcholine (10^−3^ µM) was recorded. Then, the organ was subjected to different concentrations of the decoction. The dose–response curves of the plant extract at 1, 2, and 4 mg/mL were recorded. The value of the amplitude before administration of the extracts or by the stimulation of acetylcholine was considered as a reference. The effects of the decoction on the intestinal spasms or those induced by acetylcholine were expressed as a percentage of inhibition = ((AB − AE)/AB) × 100, where AB is the average of basal tone spasm or of stimulation with acetylcholine, and AE is the average of spasms in the presence of the extract or of relaxation provoked by the extract on the contraction induced by acetylcholine [51]. Mac Ewen’s physiological solution was composed (in mM) of NaCl (130), KCl (5.63), CaCl_2_ (5.52), Na_2_HPO_4_ (0.93), NaHCO_3_ (11.9), MgCl_2_ (0.24), and glucose (11) (pH 7.4). All tests were carried out in triplicate.

### 4.15. In Vivo Antidiarrheal Assays

In vivo antidiarrheal assays were performed according to the literature with some modifications [33]. Twenty-five Wistar rats (170–230 g) were fasted for 24 h with access to water and divided into five groups of five animals. The DPBR sample at doses of 250, 500, and 1000 mg/kg of body weight was administered orally to each group. The fourth group received distilled water (negative control), while the fifth group received the standard drug loperamide at 5 mg/kg of body weight. One hour after the drug pre-treatment, all of the animals orally received 10 mL/kg of body weight of castor oil. Subsequently, each group of animals was kept separately in cages on a Whatman paper for the collection of diarrheal feces. The animals had access to water and food throughout the experiment. The severity and consistency of the diarrhea were observed hourly for 4 h after castor oil administration. The percentage of protection, inhibition of diarrhea, and humidity were calculated by the following formulas: Percentage of inhibition of diarrhea = (total number of diarrheal stools in the negative control − total number of diarrheal stools in treated group)/total number of diarrheal stools in the negative control) × 100 [52]; percentage of protection = (number of rats without diarrheal stools/total number of rats) × 100 [53]; and percentage of humidity = ((WSW − DSW)/WSW) × 100, where WSW is the wet stool weight and DSW is the dried stool weight [33,54].

### 4.16. Castor Oil-Induced Enteropooling Test

The castor oil-induced enteropooling test was performed following the methodology described by Robert et al. [55] with some modifications. Rats were deprived of food and water for 24 h to reduce food remnants from the small intestine to a minimum. Animals were randomly selected from each group of six rats. The rats were pre-treated with 100, 250, and 500 mg/kg of DPBR or 5 mg/kg of loperamide, while the control group received only distilled water solution (10 mL/kg). An amount of 10 mL/kg of castor oil was administered to each animal 30 min after DPBR or loperamide administration. Then, 30 min after the administration of castor oil, each rat was euthanized and the small intestine was tied and its weight measured. The intestinal fluid contents relative to the portion from the pylorus to the cecum were collected and placed in a 5 mL syringe whose tip was sealed. The intestines were reweighed and the differences between full and empty intestines were calculated [56]. The percentage inhibition of intestinal fluid secretion was determined as follows: % inhibition of intestinal volume = [(A − B)/A] × 100, where A is the value of intestinal fluid secretion provoked by castor oil and B indicates the value of intestinal fluid secretion after treatment with the standard drug or test substance.

### 4.17. Statistical Analysis

We used Graph Pad Prism version 8.4.3.686 (GraphPad Software Inc., San Diego, CA, USA) for statistical analyses. The results were presented as mean ± standard deviation (SD) of replication determinations according to the assay. One-way analysis of variance was used to determine the significant difference (*p* < 0.05) between concentrations. In addition, Dunnett’s multiple comparisons test and/or Sidak’s multiple comparison test was used to evaluate the difference between the treatment means. IC_50_ and EC_50_ values were calculated using nonlinear regression.

## 5. Conclusions

In this study, toxicological and pharmacological analyses showed that the decoctions of *P. butyracea* stem barks were not toxic and exhibited myorelaxant, antispasmodic, antioxidant, and antidiarrheal activities. This fact is compatible with their use in traditional medicine and emphasizes the potential of these plant extracts as a future source of new antidiarrheal drugs. Fourteen compounds were identified in *P. butyracea* stem barks, and biflavonoids (75%) were the main metabolites. Considering the previously reported vasorelaxant activities of biflavonoids [24], we thus postulate that the biflavonoids in a decoction of *P. butyracea* stem barks are responsible for its antidiarrheal activity. However, a study on the effect of pure biflavonoids has to be performed for confirmation.

## Figures and Tables

**Table 1:** List of plants selected.

Genus, Species	Family	n° NHG	Organ	Uses	Traditional Indications
*Aucoumea klaineana* Pierre	Burseraceae	599	Stem bark	Diarrhea	Macerated stem bark is used as an astringent antidiarrheal agent
*Pentadesma butyracea* Sabine	Clusiaceae	14,802	Stem bark	Diarrhea	In the decoction, stem bark is used as an antidiarrheal agent
*Canarium schweinfurthii* Engl.	Burseraceae	1724	Stem bark	Pain	In the decoction, the stem bark is used for stomach and intestinal pains
*Scorodophloeus zenkeri* Harms	Fabaceae	1418	Stem bark	Constipation	An infusion of stem bark is used to treat constipation

NHG: National Herbarium of Gabon.

**Table 2:** Polyphenols and flavonoids contents in decoctions of *P. butyracea* stem barks.

Extract	Total Phenolic Content as µg GAE/mg ± SD ^a^	Total Flavonoid Content as µg QE/mg ± SD ^a^
DPBY	62.1 ± 1.4	41.9 ± 4.5
DPBR	44.1 ± 2.9	83.0 ± 9.3
DPBD	129.0 ± 10.3	150.3 ± 30.3
*p*-value	<0.001	<0.001
R^2^	0.9916	0.9517

^a^ SD: standard deviation of three independent experiments. GAE: gallic acid equivalent; QE: quercetin equivalent.

**Table 3:** Metabolite annotation by UHPLC-ESI-MS/MS analysis of the DPBR sample. Exp and Calc *m*/*z*: experimental and calculated *m*/*z* values.

n°	RT min	[M − H]^− a^ [M + HCOO]^− b^		[M + H]^+ c^ [M + Na]^+ d^	Molecular Formula	Proposed Metabolite	Fragment Ions in Negative (−) or in Positive (+) Mode
		***Exp m*** **/*z Calc m*/*z***					
**1a**	0.61	209.0302 ^a^	209.0303 ^a^		C_6_H_10_O_8_	D-Glucarate	(−) 71/85/133/191
**1b**	0.61	355.0514 ^a^	355.0518 ^a^		C_11_H_16_O_13_	Unknown glycan	(−) 59/73/87/99/115/275/337
**1c**	0.62	204.9989 ^a^	204.9992 ^a^		C_6_H_6_O_8_	Oxalomalic acid	(−) 71/99/115/143/161
**1d**	0.62	179.0561^a^	179.0564 ^a^	203.0528 ^d^	C_6_H_12_O_6_	D-Glucose	(−) 59/71/89/113
**1e**	0.63	195.0509 ^a^	195.0510 ^a^	219.0470 ^d^	C_6_H_12_O_7_	D-Gluconic acid	(−) 59/75/99/129
**1f**	0.63	193.0353 ^a^	193.0353 ^a^	217.0320 ^d^	C_6_H_10_O_7_	D-Glucuronic acid	(−) 59/71/85/99/103/131/175
**1g**	0.64	223.0452 ^a^	223.0459 ^a^		C_7_H_12_O_8_	Tetrahydroxy 2, 3, 4, 5 heptanedioic acid	(−) 59/71/73/85/103/115/133 /149/205
**1h**	0.70	369.0671 ^a^	369.0674 ^a^	393.0646 ^d^	C_12_H_18_O_13_	Unknown glycan	(−) 73/99/127/189
**1i**	0.70	105.0194 ^a^	105.0192 ^a^	129.0181 ^d^	C_3_H_6_O_4_	Glyceric acid	(−) 45/59/75
**1j**	0.71	267.0720 ^a^	267.0721 ^a^	291.0692 ^d^	C_9_H_16_O_9_	Pentahydroxy 2, 3, 4, 6, 7 nonanedioic acid	(−) 59/71/89/113/228/249
**1k**	0.71	351.0566 ^a^	351.0568 ^a^	353.0712 ^c^	C_12_H_16_O_12_	4-(4-Deoxy-beta-D-gluc-4-enuronosyl)-galacturonic acid	(−) 59/71/83/99/143/171/189
**1l**	0.74	189.0040 ^a^	189.0043 ^a^	191.0184 ^c^	C_6_H_6_O_7_	Oxalosuccinic cid	(−) 73/83/99/127/171
**1m**	0.85	133.0143 ^a^	133.0142 ^a^	157.0110 ^d^	C_4_H_6_O_5_	Malic acid	(−) 71/89/115
**1n**	0.97	267.0720 ^a^	267.0721 ^a^		C_9_H_16_O_9_	Pentahydroxy 3, 4, 5, 6, 7 nonanedioic acid	(−) 59/71/89/101/119/133/249
**2**	1.31	191.0197 ^a^	191.0197 ^a^	215.0161^d^	C_6_H_8_O_7_	Citric acid	(−) 111/173
**3**	2.36	205.0352 ^a^	205.0353 ^a^	229.0320 ^d^	C_7_H_10_O_7_	Methyl citric acid	(−) 71/87/101/125/187
**4**	3.79	153.0193 ^a^	153.0193 ^a^		C_7_H_6_O_4_	Dihydroxybenzoic acid	(−) 109
**5**	3.91	445.1348 ^a^	445.1351 ^a^	469.1317 ^d^	C_19_H_26_O_12_	Hydroxybenzoyl rhamnosylglucose	(−) 59/93/137/289/307/417
**6**	4.15	461.1299 ^a^	461.1300 ^a^	463.1439 ^c^ 485.1256 ^d^	C_19_H_26_O_13_	Dihydroxybenzoyl rhamnosylglucose	(−) 109/152
**7**	4.31	461.1662 ^a^	461.1663 ^a^	463.1827 ^c^ 485.1640 ^d^	C_20_H_30_O_12_	Verbasoside	(−) 123/153/307
**8**	4.59	387.0930 ^a^	387.0932 ^a^	389.1080 ^c^ 411.0896 ^d^	C_16_H_20_O_11_	Hydroxybenzoyl pentahydroxy 2, 3, 4, 6, 7 nonanedioic acid	(−) 59/93/113/137 /211/231/249/267
**9**	4.90	417.1035 ^a^	417.1038 ^a^	419.1197 ^c^	C_17_H_22_O_12_	Methoxyhydroxybenzoyl pentahydroxy 2, 3, 4, 6, 7 nonanedioic acid	(−) 59/71/85/113/123/167/249/267
**10**	5.17	491.1768 ^a^ 537.1822 ^b^	491.179 ^a^ 537.1824 ^b^	493.1923 ^c^ 515.1739 ^d^	C_22_H_34_O_15_	Antiarol rutinoside	(−) 89/101/125/153/163/247/307
**11**	5.75	371.0980 ^a^	371.0983 ^a^		C_16_H_20_O_10_	Benzoyl pentahydroxy 2, 3, 4, 6, 7 nonanedioic acid	(−) 59/71/85/113/121/231/249
**12**	6.13	577.1560 ^a^	577.1562 ^a^	579.1714 ^c^	C_27_H_30_O_14_	Vitexine *O*-rhamnoside	(−) 293/413/457 (+) 283/313/415/433
**13**	6.48	879.1988 ^a^	879.1989 ^a^	881.2120 ^c^	C_42_H_40_O_21_	Morelloflavone diglucoside	(−) 125/151/403/429/565/717 (+) 241/327/403/431/557/719
**14**	6.90	477.2335 ^a^ 523.2393 ^b^	477.2341 ^a^ 523.2396 ^b^		C_23_H_40_O_13_	Dimethoxyhydroxyphenyl rhamnosylglucopyranoside	(−) 59/71/101/161/301/331
**15**	7.36	717.1460 ^a^	717.1461 ^a^	719.1614 ^c^	C_36_H_30_O_16_	Fukugiside	(−) 125/151/309/403/429/565/ 591 (+) 241/327/403/431/557
**16**	8.11	701.1511 ^a^	701.1511 ^a^	703.1665 ^c^	C_36_H_30_O_15_	Spicataside	(−) 125/151/385/387/413/539 (+) 241/311/387/415/541
**17**	8.88	555.0928 ^a^	555.0931 ^a^	557.1082 ^c^	C_30_H_20_O_11_	Morelloflavone	(−) 125/151/295/401/403/429 (+) 241/327/403/431
**18**	9.55	539.0981 ^a^	539.0982 ^a^	541.1136 ^c^	C_30_H_20_O_10_	Volkensiflavone	(−) 107/125/151/385/387/413(+) 241/311/387/389/415

**Table 4:** **DPPH radical** scavenging activities of decoctions of *P. butyracea* stem barks expressed as IC_50_ values. DPBR: rainy season; DPBD: dry season; DPBY: young tree in the dry season.

**Extracts**	Free Radical Scavenging Activity IC_50_ (µg/mL ± SD ^a^)
Ascorbic acid	6.2 ± 1.2
DPBR	23.5 ± 2.1
DPBD	8.1 ± 0.6
DPBY	11.0 ± 2.0
*p*-value	0.005
R^2^	0.7838

^a^ SD: standard deviation of three independent experiments.

**Table 5:** Effect of DPBR sample’s myorelaxant and antispasmodic activities on smooth muscle.

Extracts	Concentration (mg/mL)	% Relaxation ± SD ^a^	EC_50_ (mg/mL)
DPBR	1	32 ± 5	1.6 ± 0.4
	2	68 ± 1.7	
	4	100 ± 0	
Loperamide	4	55 ± 4	
Acetylcholine (10^−3^ µM) + DPBR	1	0	
	2	30.2 ± 1.4	
	4	42.9 ± 5	

^a^ SD: standard deviation of three independent experiments.

**Table 6:** Effects of the DPBR on castor oil-induced diarrhea.

Treatment	Rats with Diarrhea/ Group	Protection (%)	Number Dried Stools	Number Wet Stools	% Inhibition of Diarrhea	WSW (g) ± SD ^a^	DSW (g) ± SD ^a^	Humidity (%) ± SD ^a^
Control	5/5	0	3	13	0	2.54 ± 0.9	0.60 ± 0.37	75.5 ± 13.5
Loperamide (5 mg/kg)	3/5	40	11	7	46.2	2.20 ± 0.9	0.96 ± 0.27	48.4 ± 13.5 ^α;^ **
DPBR (100 mg/kg)	3/5	40	9	7	46.2	2.08 ± 0.7	0.64 ± 0.17	68.36 ± 4.3
DPBR (250 mg/kg)	2/5	60	10	2	84.6	1.43 ± 0.65	0.57 ± 0.32	54.3 ± 12 ^α;^ *^; β; ns^
DPBR (500 mg/kg)	0/5	100	8	0	100	1.32 ± 1.1	0.84 ± 0.50	39 ± 7.8 ^α;^ ***^; β; ns^
DPBR (1000 mg/kg)	3/5	40	9	9	30.8	4.12 ± 0.9	1.65 ± 0.27	54.8 ± 10.1

^a^ SD: standard deviation of three independent experiments. WSW: wet stool weight; DSW: dried stool weight. (WSW − DSW)/WSW) × 100. Data were collected 4 h after the administration of samples or water. One-way analysis of variance (ANOVA) followed by Dunnett’s multiple comparison test was used to assess differences between groups. A value of *p* ˂ 0.05 was considered statistically significant. * *p* < 0.01; ** *p* < 0.001; *** *p* < 0.0001. ^α^ compared to water; ^β^ compared to loperamide; ns: no significant.

**Table 7:** Effect of *p. butyracea* stem bark decoction DPBR on castor oil-induced enteropooling in rats.

Group	Dose (mg/kg)	Weight of Intestinal Content (g)	Percent Inhibition Weight (%)	Volume Intestinal Fluid (mL)	Reduction in Volume of Intestinal Content (%)
Control	-	3.2 ± 0.2	-	2.9 ± 0.13	-
Loperamide	5	1.5 ± 0.1 ****	51.9	1.0 ± 0.03 ****	63.8
DPB	100	2.7 ± 0.2 *	12.5	2.3 ± 0.2 *	20.7
	250	2 ± 0.2 ***	36.5	1.75 ± 0.2 ****	39.7
	500	1.1 ± 0.24 ****	64.7	0.45 ± 0.06 ****	84.5

* *p* < 0.05; *** *p* < 0.001; **** *p* < 0.0001 compared with the negative control vs. loperamide and DPBR for weight of intestinal content or volume of intestinal fluid with one-way ANOVA followed by Dunnett’s multiple comparisons (*n* = 6).

## Data Availability

All data are presented in the article.

## Supplementary Materials

The following supporting information can be downloaded at: https://www.mdpi.com/article/10.3390/molecules29235789/s1, Figure S1: EI-MS (a) and ESI-MS/MS (b) spectra of pentahydroxy 2, 3, 4, 6, 7 nonanedioic acid. For GC-MS analysis, the diacid was analyzed as a methylester trimethylsilyl (TMS) derivative. (c) ESI-MS/MS spectrum of [M − H]^−^ at *m*/*z* 387.093 of 2-hydroxybenzoyl pentahydroxy 2, 3, 4, 6, 7 nonanedioic acid; Figure S2: Survival percentage of two human cell lines, HEK-293 and hCMEC/D3, incubated with 100 µg/mL of DPBR; Figure S3: Body weight gain of the Wistar rats during 14-day period after treatment with 2000 mg/kg of DBPR compared to the control group. Table S1: Metabolite annotation by GC-MS analysis of the DPBR sample after methanolysis and trimethylsilylation. The percentage of each metabolite was determined on the basis of the relative ratio in GC-EI-MS of the corresponding peak area and relative response factors determined on standard monosaccharides. (*) selective fragment ions.
