# Supplementary material for: Phytochemical Analysis and Antidiarrheal Activity of Stem Bark Decoctions of Pentadesma butyracea Sabine (Clusiaceae)
Source: Molecules. 2024 Dec 7;29(23):5789. doi: 10.3390/molecules29235789 (PMC11643820; doi:10.3390/molecules29235789)
Supplement: Supplementary file 1 [file molecules-29-05789-s001.zip › molecules-3228536-supplementary.pdf]

Supplementary Material: Table

Table S1. Metabolite annotation by GC-MS analysis of the DPBR sample after methanolysis and trimethylsilylation. Percentage of each metabolite was determined on the basis of the relative ratio in GC-EI-MS of the corresponding peak aera and relative response factors determined on standard monosaccharides.

**Compound**	**Fragment ions in EI-MS**	**Relative abundance (%)**
Arabinose (Ara)	204/217/333	11 ± 1.5
Rhamnose (Rha)	204/217/305	2 ± 0.5
Fucose (Fuc)	204/217/305	< 1
Xylose (Xyl)	204/217/333	5 ± 0.8
Galacturonic acid (GalA)	204/217/391	3 ± 0.5
Mannose (Man)	204/217/377	1 ± 0.3
Galactose (Gal)	204/217/377	7 +/− 1
Glucose (Glc)	204/217/377	68 ± 8
Gluconate/Galactonate	59/103/205/217275/319/377	2 ± 0.5
Hydrobenzoic acid	73/193/224	< 1
Dihydroxybenzoic acid	73/193/312	< 1

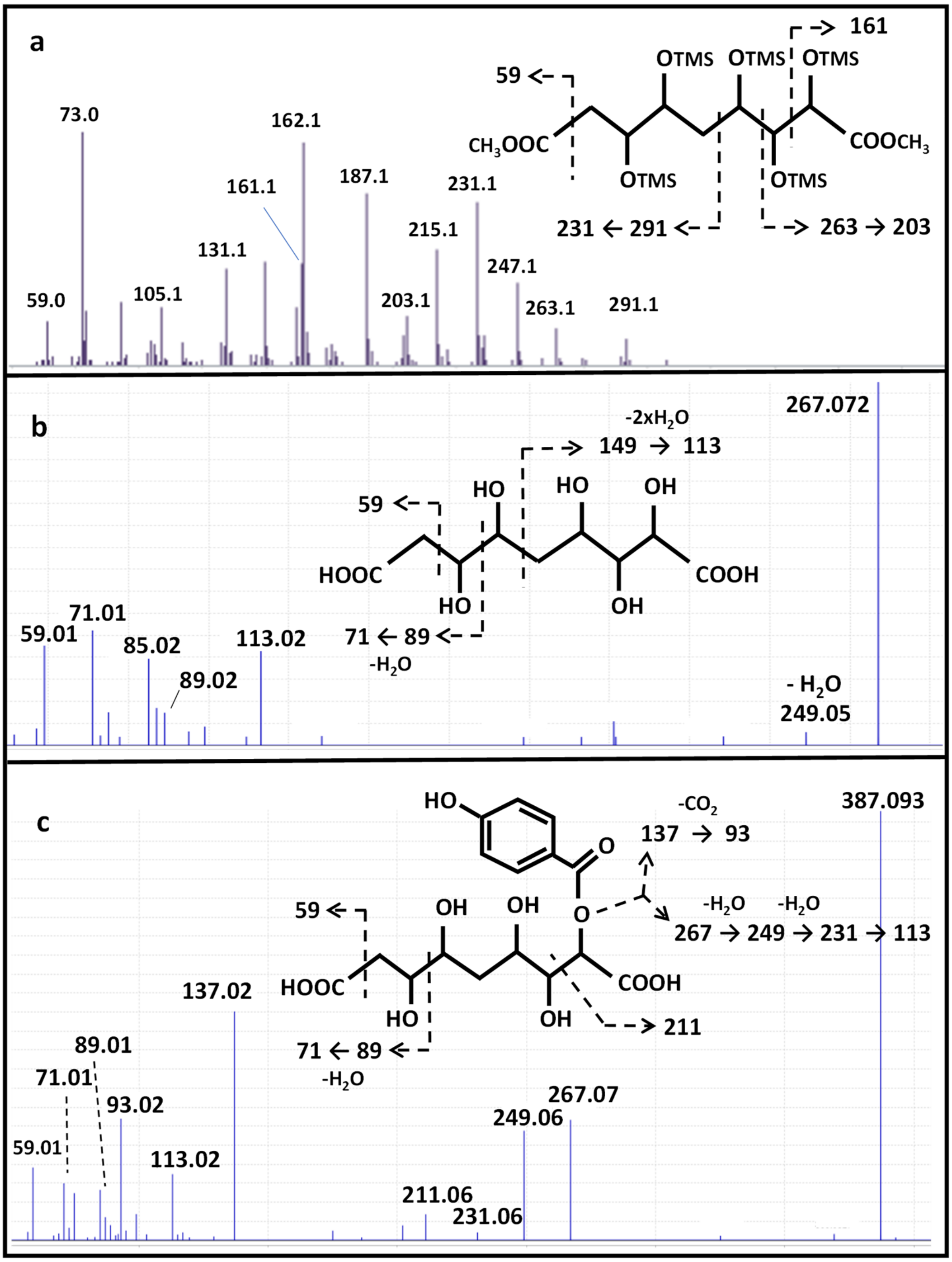

Figure S1. EI-MS **(a)** and ESI-MS/MS **(b)** spectra of pentahydroxy 2, 3, 4, 6, 7 nonanedioic acid. For GC-MS analysis, the diacid was analysed as a methylester trimethylsilyl (TMS) derivative. **(c)** ESI-MS/MS spectrum of $[M-H]^-$ at *m/z* 387.093 of 2-hydroxybenzoyl pentahydroxy 2, 3, 4, 6, 7 nonanedioic acid.

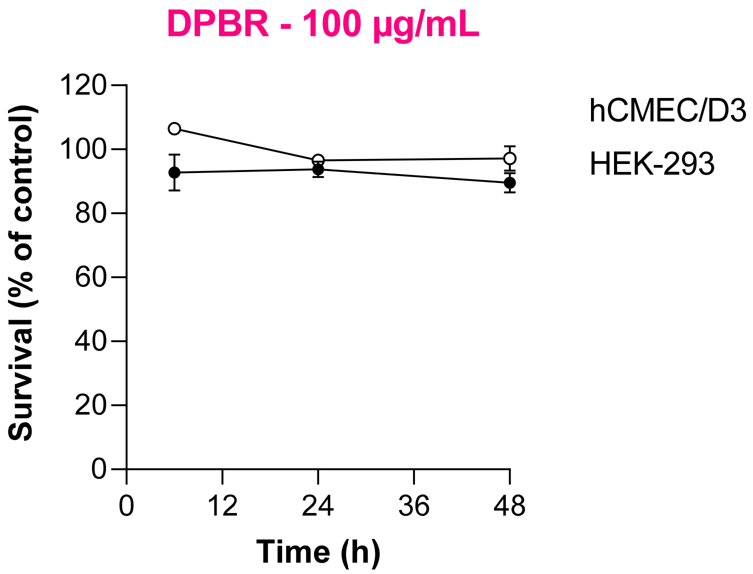

Figure S2. Survival percentage of two human cell lines, HEK-293 and hCMEC/D3, incubated with 100 µg/mL of DPBR.

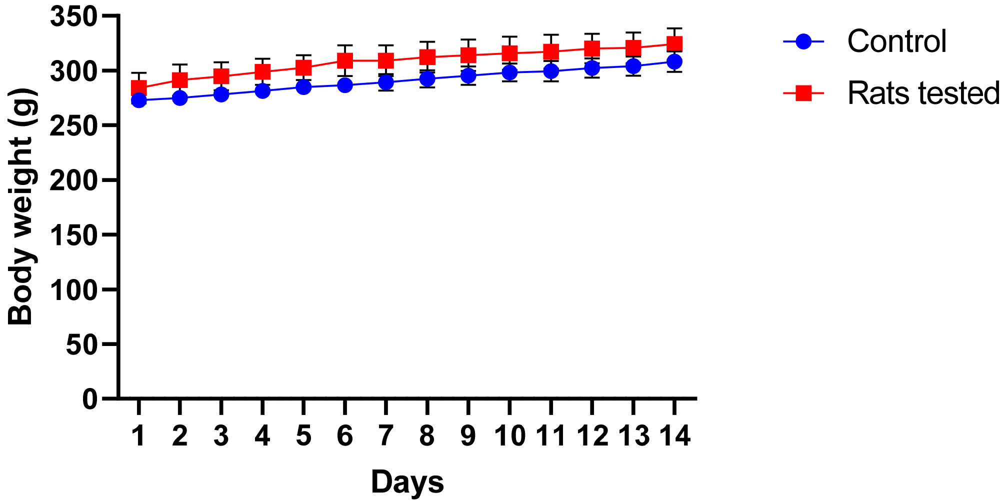

Figure S3. Body weight gain of the Wistar rats during a 14-day period after treatment with 2000 mg/kg of DBPR compared to the control group.
